# Antimalarial pharmacology and therapeutics of atovaquone

**DOI:** 10.1093/jac/dks504

**Published:** 2013-01-04

**Authors:** Gemma L. Nixon, Darren M. Moss, Alison E. Shone, David G. Lalloo, Nicholas Fisher, Paul M. O'Neill, Stephen A. Ward, Giancarlo A. Biagini

**Affiliations:** 1 Liverpool School of Tropical Medicine, Pembroke Place, Liverpool L3 5QA, UK; 2 Department of Chemistry, Liverpool University, Liverpool L69 7ZD, UK

**Keywords:** malaria, drug development, mechanism of action, resistance, drug interactions

## Abstract

Atovaquone is used as a fixed-dose combination with proguanil (Malarone) for treating children and adults with uncomplicated malaria or as chemoprophylaxis for preventing malaria in travellers. Indeed, in the USA, between 2009 and 2011, Malarone prescriptions accounted for 70% of all antimalarial pre-travel prescriptions. In 2013 the patent for Malarone will expire, potentially resulting in a wave of low-cost generics. Furthermore, the malaria scientific community has a number of antimalarial quinolones with a related pharmacophore to atovaquone at various stages of pre-clinical development. With this in mind, it is timely here to review the current knowledge of atovaquone, with the purpose of aiding the decision making of clinicians and drug developers involved in the future use of atovaquone generics or atovaquone derivatives.

## Introduction

Atovaquone is the end product of half a century of research by many groups who researched the antiparasitic properties of numerous structurally related compounds.^1–6^ Currently, atovaquone is used as a fixed-dose combination with proguanil (Malarone) for the treatment of children and adults with uncomplicated malaria or as a chemoprophylactic agent for preventing malaria in travellers.^7,8^ In the USA, between 2009 and 2011, Malarone accounted for 70% of all antimalarial pre-travel prescriptions.^9^

**Figure 1.**
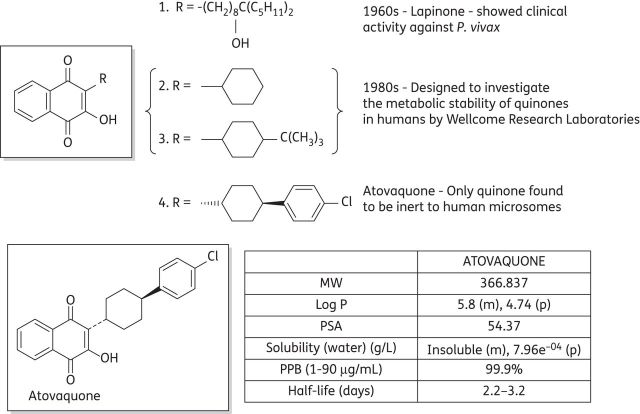
Historical development of atovaquone and its pharmacokinetic properties. MW, molecular weight; m, measured; p, predicted; PSA, polar surface area; PPB, plasma protein binding.

The development of atovaquone as an antimalarial drug began more than 50 years ago when the outbreak of World War II caused substantial shortages in the supply of quinine.^10^ Intense efforts in the USA led to thousands of structurally diverse compounds being investigated, several of which were hydroxynaphthoquinones. Modest antimalarial activity when administered to ducks infected with *Plasmodium lophurae* resulted in a robust optimization programme generating more than 300 quinones, some of which demonstrated greater activity than quinine in the duck assay. However, when administered to malaria patients these compounds were devoid of any activity due to poor absorption and rapid metabolism.^11,12^ Attempts to solve these problems and produce an orally active quinine were unsuccessful both then and when the problem was revisited in the 1960s.^13^ Research in the 1960s did, however, lead to the development of lapinone (1), which was given intravenously and had activity against *Plasmodium vivax* (Figure 1).^14^

**Figure 2.**
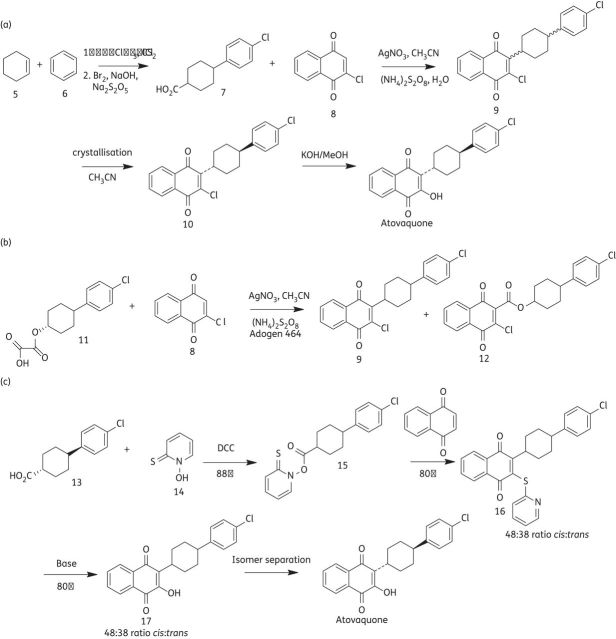
Synthetic routes used to synthesize atovaquone. (a) The original synthesis of atovaquone. (b) Williams and Clarke atovaquone synthesis. (c) Improved atovaquone synthesis.

The use of quinones as antimalarial agents was then reinvestigated in the 1980s by a group at the Wellcome Research Laboratories. More meaningful studies could be carried out at this time due to the development of test systems using the human parasite *Plasmodium falciparum in vitro* or in *Aotus* monkeys. The aim of this study was to design a quinone with good antimalarial activity against *P. falciparum* combined with good metabolic stability in humans. Several 2-cyclohexyl-3-hydroxy-1,4-naphthoquinone analogues (2 and 3) were synthesized with the metabolically labile 4′ position of the cyclohexyl ring substituted with a range of groups.^15,16^ Several of these quinones demonstrated a potency of ∼1 nM towards *P. falciparum in vitro*, but only atovaquone (4) was inert to human liver microsomes.^17,18^ The *trans* isomer of atovaquone is substantially more potent than the corresponding *cis* isomer. The chemical synthesis of atovaquone was originally disclosed in 1991 in US patent no. 4981874. This route gave a poor yield of 4% atovaquone calculated from only the last two steps (Figure 2a).^19^

Williams and Clark^20^ then published a variant of this methodology (Figure 2b) in which oxalate (11) was used to produce racemic compound (9) with a 43% yield and the ester by-product (12) with a 38% yield. Conversion into atovaquone was then achieved as described in Figure 2(a). The disadvantages of this process are the column chromatography required to separate (9) from (12) and the same poor yield problem still prevails in the final two steps.

Both processes described so far also involve the use of silver nitrate, a heavy metal that can be difficult to remove and whose use is tightly regulated. The recently patented (WO 2010/001379) synthesis seen in Figure 2(c) offers an improved synthesis of atovaquone, as it is higher yielding and does not involve the use of heavy metals.^21^

A common problem with all the routes so far is that large amounts of the potentially useful, yet significantly less potent *cis* isomer of atovaquone are disregarded, as only the *trans* isomer is required. There are two literature procedures that address this problem. Reacting the *cis* isomer of atovaquone, atovaquone intermediates or isomeric mixtures thereof with a strong acid results in a clean epimerization to the corresponding *trans* isomer and thus high yields of *trans* atovaquone.^22^ Heating the *cis* isomer at reflux in organic solvent also causes this transformation.^23^

With the patent relating to Malarone due to expire in 2013, the synthesis of atovaquone will be exploited to its full potential as generic versions of the drug are likely to become commonplace. This will in turn have a marked effect on the cost, as currently the high cost of atovaquone is frequently prohibitive in its use by the endemic population within countries affected by malaria. Increased availability and use of the drug will also have an effect on the clinical efficacy of atovaquone, and factors such as access, sustainability and resistance need to be considered.^24^ Furthermore, the malaria scientific community has a number of antimalarial quinolones with a pharmacophore related to atovaquone at various stages of pre-clinical development.^25–30^

## Pharmacodynamics

### Mode of action

Atovaquone is a competitive inhibitor of ubiquinol, specifically inhibiting the mitochondrial electron transport chain at the *bc*_1_ complex.^31^ Inhibition of *bc*_1_ activity results in a loss of mitochondrial function.^32,33^ During the intra-erythrocytic stage of infection, a key role of the parasite mitochondrion is to provide orotate for pyrimidine biosynthesis through the activity of dihydroorotate dehydrogenase (DHODH). Consistent with this, inhibition of the *bc*_1_ complex by atovaquone affects the concentrations of metabolites in the pyrimidine biosynthetic pathway.^34,35^ Indeed, transgenic *P. falciparum* parasites expressing ubiquinone-independent yeast DHODH have been shown to display an atovaquone-resistant phenotype.^36^ In addition, a recent study suggests that a further cellular consequence of mitochondrial inhibition by atovaquone is the inhibition of purine biosynthesis.^37^ Blood-stage parasite death as a result of atovaquone is relatively slow compared with other antimalarials such as artemisinin and chloroquine.^25,38^ This feature appears to be consistent with other mitochondrial-acting antimalarials and is possibly due to the drug acting only on late trophozoites and not on the earlier ‘ring’ stages.^25^ Atovaquone is, however, active against liver stages, resulting in its utility as a prophylactic drug; however, it is not believed to be active against ‘dormant’ hypnozoites.^8,39^

### Mechanism of parasite resistance to atovaquone/malarone

**Figure 3.**
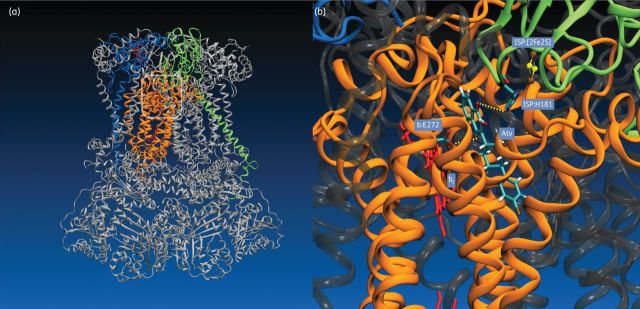
(a) Cartoon representation of the yeast cytochrome *bc*_1_ complex (3CX5.PDB), with atovaquone modelled at the Q_o_ site (boxed area).^83^ The *bc*_1_ complex is a structural and functional homodimer with a molecular mass of ∼480 kDa, consisting of 10 discrete subunits per monomer in yeast and *P. falciparum*.^28^ The electron-transferring catalytic unit of one monomer is highlighted; cytochrome *b* is represented in orange, cytochrome *c*_1_ in blue and the Rieske iron-sulphur protein (ISP) in green. Haem groups (cyt *b* and cyt *c*_1_) are shown in red. The remaining subunits of the complex are rendered in grey. (b) Molecular model of atovaquone (Atv) bound to the Q_o_ site of the *bc*_1_ complex. Subunits are coloured as in panel (a). Atovaquone was modelled into the Q_o_ site of cytochrome *b* as described by Fisher *et al*.^46^ Hydrogen-bonding interactions between the naphthoquinone head group of atovaquone and side chains of Glu-272 (cyt *b*) and His-181 (ISP) are indicated by yellow lines. The positions of haem *b*_l_ (cyt *b*) and the ISP [2Fe2S] cluster are also shown.

Although the crystal structure of the *P. falciparum* cytochrome *bc*_1_ complex is not available, details of atovaquone binding to cytochrome *b* have been elucidated based on studies performed on model organisms and molecular modelling. These studies, which include electron paramagnetic resonance spectroscopy of the Rieske [2Fe2S] cluster, site-directed mutagenesis of model organism cytochrome *b* and gene sequencing of atovaquone-resistant *Plasmodium* species, demonstrate that atovaquone is most likely a competitive inhibitor of the parasite's cytochrome *b* quinol oxidation (Q_o_) site (Figure 3).^28,40^

Malarone drug failure has been associated with a missense point mutation at position 268 in cytochrome *b*, exchanging tyrosine for serine (Y268S) or, less frequently, asparagine (Y268N).^41–45^ Position 268 in cytochrome *b* is highly conserved across all phyla and is located within the ‘ef’ helix component of the Q_o_ site, which is putatively involved in ubiquinol binding. The resultant atovaquone-resistant growth IC_50_ (half-maximal inhibitory concentration) phenotype of these mutants is some 1000-fold higher than susceptible strains; however, this is accompanied by an ∼40% reduction in the *V*_max_ of the *bc*_1_ complex, suggestive of a significant fitness cost to the parasite.^46^

It is well documented that atovaquone monotherapy gives rise to *de novo* resistance very rapidly.^47,48^ However, the underlying reason for this phenomenon has not been determined and, as discussed in the next section, may be partially explained by pharmacodynamic/pharmacokinetic considerations (related to the physicochemical properties of atovaquone combined with a slow rate of sterilization) as well as hitherto untested considerations related to the molecular target such as, e.g. the effect of an increased mutation rate of mitochondrially encoded genes such as cytochrome *b* compared with nuclear encoded genes.^49^

Furthermore, it has been reported that an *in vitro* atovaquone-resistant parasite line has been generated in the laboratory possessing wild-type cytochrome *b*.^50^ The mechanism underpinning the parasite's atovaquone-resistant phenotype in this strain remains to be elucidated.

The speed of development of resistance to a new antimalarial is an important consideration. According to the Medicines for Malaria Venture (MMV) target product profiles (TPPs), pre-clinical development of new *bc*_1_-acting antimalarials must show activity against a panel of multidrug-resistant antimalarial parasites that include atovaquone-resistant isolates. There are also *in vitro* speed of development of resistance assays that are available that can be used to guide go/no-go development decisions.^51^ Whether the observed rapid onset of *de novo* resistance seen in atovaquone is based on the physicochemical property of the molecule or whether it is based on inherent issues relating to the biological target, it is likely that new *bc*_1_-target antimalarials will require marriage with a partner drug, unless the candidate drugs possess biologically distinct polypharmacology.

### Pharmacokinetics

**Figure 4.**
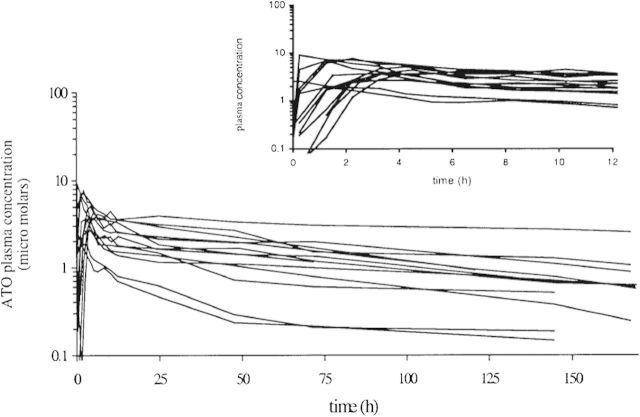
Atovaquone plasma concentration–time profile after a single dose of Malarone in 13 healthy individuals. Reproduced with permission from the study by Thapar *et al*.^52^

The pharmacokinetic parameters of atovaquone in the currently utilized formulation (Malarone, 250 mg atovaquone + 100 mg proguanil) have been determined (Figure 4).^52^ The median atovaquone plasma AUC (h/μM), *t*_1/2_ (h), *C*_max_ (μM) and *T*_max_ (h) were 295, 87.2, 3.74 and 3.25, respectively, following a single dose and 254, 55.9, 13.8 and 4.00, respectively, upon reaching steady state. The similar AUC values observed between single-dose and steady-state dosing suggest no unexpected accumulation of atovaquone following repeated administration, although this may be due to saturation of plasma atovaquone concentrations, and an increase in atovaquone concentrations in tissues cannot be ruled out.

Atovaquone IC_50_ against susceptible malaria *in vitro* is very low, ranging from 1 to ∼3.5 nM.^31,53,54^ This has resulted in the belief that atovaquone plasma concentrations (around 1–10 μM; see Figure 4) are sufficient to produce total suppression of malaria. However, atovaquone shows extremely high levels of plasma protein binding (>99.5%) and therefore the concentration of unbound atovaquone is likely to be significantly lower.^55^ Extrapolations of pharmacokinetic/pharmacodynamic dynamics using *in vitro* data should therefore be treated with caution.

At present, there are no established minimum effective plasma concentrations of atovaquone for malaria prophylaxis. However, a clear correlation between atovaquone steady-state plasma concentration and treatment success has been established in *Pneumocystis* pneumonia in patients with AIDS.^56^ Atovaquone plasma concentrations of 10 to <15 μg/mL and 15 to <20 μg/mL resulted in 79% and 95% treatment success, respectively. Furthermore, there have been case reports of atovaquone treatment failure in antimalarial therapy that were not explained by drug resistance mutations, and patients with a body weight >100 kg have a marked increased chance of treatment failure compared with <100 kg patients, both of which suggest drug concentration may be a factor in determining treatment failure.^42,57,58^ The prediction of atovaquone therapy failure and resistance selection using drug concentration parameters has the potential to improve current patient therapy and an investigation determining a pharmacokinetic/pharmacodynamic relationship is warranted.

### Absorption

Absorption of atovaquone shows dose limitations, with maximum absorption observed using 750 mg tablets.^59^ Poor drug solubility was suggested as the cause of this limit to absorption, and this led to the development of an atovaquone liquid suspension formulation that showed improved *Pneumocystis* pneumonia treatment success compared with the tablet formulation.^60^

The bioavailability of 750 mg atovaquone when taken with food was 23% in HIV-infected patients.^61^ Combining data from six clinical trials, the interpatient variability of atovaquone bioavailability is substantial and has been determined to be 107%, which is likely due to the drug's low solubility and the effects of food.^61–63^

The oral absorption of atovaquone increased when taken with a high-fat meal (two slices of toast with 56 g of butter, with 3.9-fold exposure compared with fasting), whereas a minimal-fat meal (two slices of toast) had minimal impact on absorption.^63^ Consequently it is recommended that atovaquone be taken with a high-fat meal. However, a recent *in vitro* study showed that the atovaquone IC_50_ increased 20-fold when serum used in the assay was taken from a subject recently given a high-fat meal compared with serum from a fasting subject (0.5–12 ng/mL, *P* < 0.01).^64^ A correlation between high serum triglyceride concentrations and high atovaquone IC_50_ was observed, suggesting reduced free (unbound) atovaquone concentrations due to increased drug–fat binding. The clinical relevance of this finding is unknown, but the impact to atovaquone pharmacokinetics is likely to be transient and is unlikely to outweigh the benefit of increased atovaquone absorption.

Dissolution of atovaquone tablets increases in the presence of milk, and therefore the presence of milk in meals may increase atovaquone bioavailability in patients.^62^ This may provide an alternative strategy to high-fat meals when aiming to maximize the bioavailability of atovaquone, although this has not been shown clinically.

### Distribution

Atovaquone is highly bound to plasma protein (>99.5%) and shows a high affinity for human serum albumin, although the low drug clearance rate suggests that atovaquone may also accumulate in tissues, where it is protected from biliary clearance.^55^ In a study of atovaquone population pharmacokinetics, the volume of distribution of atovaquone was 7.98 L/kg, although individual values were markedly linked to body weight; the volume of distribution shows a linear increase with increased patient body weight.^61^

### Metabolism

Under normal conditions, there is no evidence that atovaquone is significantly metabolized in humans, or that metabolism is required for drug elimination. It may be possible that certain enzymes could be induced and therefore lead to increased atovaquone biotransformation, but this has not been demonstrated.

### Elimination

Atovaquone pharmacokinetics are characterized by an extremely long elimination half-life of ∼50–84 h.^59,63,65^ Elimination is primarily via the liver, with almost undetectable amounts (<0.6%) of drug being eliminated via the kidneys.^66^ More than 90% of the drug excreted in bile was in the parent form. Elimination of atovaquone is complicated by the possibility of enterohepatic recirculation of the drug, which may help explain atovaquone pharmacokinetic profiles where a reduction and then an increase in drug concentration is seen with time.

In a study of atovaquone population pharmacokinetics, the oral clearance of atovaquone was increased in patients with higher body weights, with 60% increased clearance seen in an 80 kg patient compared with a 40 kg patient.^61^ In the same study, the average oral clearance of atovaquone was higher in Oriental (8.49 L/h) and Malay (9.13 L/h) subjects compared with White (1–7.6 L/h) subjects.^61^

### Drug interactions

Atovaquone is highly bound to plasma protein (>99.5%) and shows a high affinity for human serum albumin.^55^ Furthermore, the half-life of atovaquone is long, ranging from ∼50 to 84 h, and the major limiting factor to atovaquone clearance is probably its plasma protein binding.^59,63,65^ This suggests that any drug that reduces atovaquone plasma protein binding may potentially alter atovaquone tissue distribution and/or clearance. However, the authors can find no published articles investigating the drug-mediated displacement of atovaquone from plasma protein and the clinical impact of these interactions, and this area requires further research. The interaction observed between atovaquone and antiretrovirals, where efavirenz, lopinavir and ritonavir (all highly protein-bound drugs) reduced atovaquone plasma concentrations in HIV-infected patients, may involve atovaquone plasma protein displacement, although this was not demonstrated.^67^ This emphasizes the importance of establishing the interactions between antimalarials, including atovaquone, and antiretrovirals.

The potential for atovaquone to displace other protein-bound drugs has been investigated. A case study was recently published that describes a potential interaction between the anticoagulant drug warfarin and atovaquone, where the author suggests that atovaquone caused an increase in free warfarin concentrations to super-therapeutic levels.^68^ A separate investigation found that atovaquone did not alter the pharmacokinetics of the antiepileptic drug phenytoin, another highly protein-bound drug, which is susceptible to displacement interactions.^69^ The evidence that atovaquone can compete with other drugs for plasma protein binding is lacking, although further investigations are required to fully understand this potential factor in atovaquone pharmacokinetics.

Atovaquone exposure is markedly decreased when taken concomitantly with the antibiotic drug rifampicin and therefore co-administration of atovaquone and rifampicin is not recommended.^70^ The mechanism behind this interaction is not fully understood, although the ability of rifampicin to induce activity in metabolism enzymes and drug transporters is assumed to be responsible. However, no metabolite of atovaquone has been identified in humans, and the impact of individual enzymes and transporters on atovaquone disposition is unclear.

There is evidence that atovaquone can inhibit cytochrome P450 enzymes, although data have been generated *in vitro* and the relevance to clinical drug interactions is unknown. Atovaquone inhibited the metabolism of 50 μM of 7-benzyloxy-4-(trifluoromethyl)-coumarin (BFC) by recombinant CYP3A4, with an IC_50_ of 4.7 μM.^52^ Similarly, sulfamethoxazole metabolism by recombinant CYP2C9 was inhibited by atovaquone, with an inhibition constant (K_i_) of 15 μM.^71^ However, when atovaquone was pre-incubated with human serum and centrifuge filtered to remove protein before use, no CYP2C9 inhibitory activity was observed. A recent case study described an HIV-infected female with a marked increase in plasma concentrations of the antiretroviral drugs etravirine (+55%) and unboosted saquinavir (+274%) following atovaquone/proguanil prophylaxis.^72^ In the same study, raltegravir plasma concentrations were unchanged following atovaquone/proguanil prophylaxis. The evidence that atovaquone/proguanil prophylaxis increases exposure of etravirine and saquinavir (both cytochrome P450 substrates) but not raltegravir (no affinity for cytochrome P450 enzymes) suggests atovaquone, proguanil or both drugs may be inhibiting cytochrome P450 activity.^73–75^

Co-administration of atovaquone and the nucleoside reverse transcriptase inhibitor zidovudine increased the exposure (33% increase in AUC_0–8_, *P* < 0.05) and decreased the oral clearance (25% reduction, *P* < 0.05) of zidovudine in HIV-infected patients.^76^ Furthermore, patients taking atovaquone showed a trend towards lower zidovudine/glucuronide plasma concentrations (6% reduction in AUC_0-8_, *P* < 0.1) and a significant decrease in the ratio between zidovudine/glucuronide and plasma concentrations (30% reduction, *P* < 0.05). Atovaquone exposure was unchanged when co-administered with zidovudine.

The atovaquone-mediated 33% increase in zidovudine exposure is itself unlikely to cause increased haematological toxicity, although caution is advised in patients taking additional drugs with similar toxicity profiles to zidovudine.^76^ Also, increased zidovudine plasma concentrations and reduced zidovudine glucuronidation may potentially lead to increased formation of the cytochrome P450-mediated zidovudine metabolite 3′-amino-3′-deoxythymidine, which shows a 7-fold higher toxicity in bone marrow cells compared with the parent drug.^77^

The increased exposure and decreased clearance of zidovudine suggests that atovaquone is inhibiting the glucuronidation of zidovudine. The primary enzyme involved in zidovudine glucuronidation is uridine 5′-diphospho-glucuronosyltransferase (UGT) 2B7.^78^ Therefore, clearance of UGT2B7 substrates, such as the anti-HIV drug efavirenz, may also be influenced by atovaquone, and further investigations are warranted in this area.^78^

Atovaquone did not alter the exposure of the anti-HIV protease inhibitor drug indinavir in healthy volunteers.^79^ Indinavir is a substrate of the drug efflux transporter ABCB1, and the absence of any effect of atovaquone on indinavir pharmacokinetics suggests that atovaquone is not altering the activity of ABCB1, although this has not been confirmed.^80^

### Safety and toxicology

Atovaquone has been found to be generally well tolerated and causes few side effects. Adverse events are generally mild and include rash, fever, vomiting, diarrhoea, abdominal pain and headache. Indeed, overdoses as large as 31 500 mg have been reported to cause little or no symptomatology.^81^

A significant concern for the development of novel antimalarials targeting the parasite *bc*_1_ is host mitochondrial toxicity. In animal models this manifests itself as acute toxicity (presumed to be cardiotoxicity). Current development projects use *in vitro* counter-screens such as human *bc*_1_ screening or human cell lines grown on galactose, making these cells more reliant on mitochondrial metabolism by circumventing the Crabtree effect.^82^ However, these projects are hampered by the absence of industry standards relating to pre-clinical or clinical mitochondrial toxicity.

### Conclusions

Despite the extensive use of atovaquone/proguanil, there remains a considerable knowledge gap concerning its pharmacology. The rollout of generics following the expiration of this patent will undoubtedly see an increase in atovaquone/proguanil usage that will be closely followed by an increase in treatment failures. Clearly, if the community is to manage this issue and develop improved derivatives, more effort needs to be directed towards understanding the pharmacokinetic/pharmacodynamic mechanisms underpinning atovaquone/proguanil treatment failure.

## Transparency declarations

None to declare.
